# Availing health services—A study on social barriers and perceptions among women of reproductive age group in Bankura District, West Bengal, India

**DOI:** 10.3389/fnut.2025.1720962

**Published:** 2025-12-18

**Authors:** Samraggi Roy, Ebbie Thomas, Mahalingam Govindaraj, Sirimavo Nair

**Affiliations:** 1 Department of Foods and Nutrition, Faculty of Family and Community Sciences, The Maharaja Sayajirao University of Baroda, Vadodara, India; 2 Department of Community Medicine, GMERS Medical College, Gotri, Vadodara, India; 3 HarvestPlus Program, Alliance of Bioversity International and the International Center for Tropical Agriculture (CIAT), Cali, Colombia

**Keywords:** anemia control program, iron and folic acid supplementation, reproductive health, gender disparities in healthcare, public health nutrition, maternal and child health, focus group discussions, nutrition-sensitive interventions

## Abstract

Despite advancements in health and technology, anemia continues to be a significant health concern for pregnant and lactating women in low- and middle-income countries, contributing to both morbidity and mortality. This study aims to explore the behavioral aspects and societal influences that affect adherence to health services and the adoption of healthy nutritional habits among women of reproductive age. Focus group discussions (FGDs) were conducted among Pregnant women, lactating mothers, and their respective mothers-in-law in four Gram Panchayats of Bankura District, West Bengal, India. The qualitative data from six focus group discussions (FGDs) were analyzed using thematic analysis in NVivo version 14.23.3. To develop thematic elements relevant to program objectives, a process of inductive coding was undertaken to support coding and creation of major themes. In addition, theme and code coverage visualizations assisted in identifying barriers, along with participant quotes and excerpts noted verbatim. The study identifies key barriers to compliance with anemia control programs, including inadequate consumption of iron and folic acid-rich foods, dislike for Iron and Folic Acid (IFA) tablets, forgetfulness, and limited awareness of anemia. Service gaps such as insufficient counseling on anemia, inadequate supply of IFA tablets, and health infrastructure issues exacerbate the problem. The focus group discussions revealed the behavioral and societal influence affects lack of adherence to services. These women portray lack of compliance with IFA tablets due to their dislike of the taste of the tablets and forgetting to take them due to increased household work, along with their aversion towards green-leafy vegetables. Additionally, these women claimed that there is an inadequate supply of IFA tablets for many lactating mothers as well as women in the reproductive age group (15–49 years of age). Gender disparities further compound these challenges, with unequal distribution of household labor and limited access to education for women contributing to nutritional deficiencies. Addressing these multifaceted issues requires comprehensive interventions focusing on nutrition education, healthcare access, and gender equality to effectively combat anemia among vulnerable populations.

## 1 Introduction

The burden of anemia among women of reproductive age (15–49 years of age) in low- and middle-income countries (LMICs) continues to be an intractable problem of “hidden hunger”, exemplifying gender health inequities and a loss of human capital (1–3). Over and above, severe anemia in pregnancy is consistently linked to maternal mortality. Micronutrient deficiencies threaten women's economic productivity, health, and even their lives. The burden of anemia among women of reproductive age (15–49 years of age) in LMICs continues to be a serious concern of “hidden hunger”, expanding gender health inequities and a loss of human capital (2, 4).

Global disparities in anemia highlight the stark differences between developing and developed nations, particularly in access to healthcare, nutrition, and basic amenities. In resource-limited settings, societal and environmental factors exacerbate health inequities, making anemia prevention and management even more challenging (5). In LMICs, inadequate healthcare services, poor dietary intake, and limited access to iron-folic acid (IFA) supplementation contribute to persistently high anemia prevalence (6). The consequences extend beyond individual health—poor maternal nutrition cascades down through generations, affecting children's growth, cognitive development, and long-term economic opportunities, further straining households, and communities.

India's public health strategy, Anemia Mukt Bharat, mandates all states to follow a comprehensive, rights-based approach for universal access to anemia prevention and treatment services. The program's 6 × 6 × 6 model integrates behavioral change, healthcare delivery, and multi-sectoral coordination, emphasizing compliance with iron-folic acid (IFA) supplementation and fortified foods through social safety net programs (12–14).

West Bengal's NFHS-5 data shows that 67% of women of reproductive age in Bankura and 70.8% of adolescent girls statewide are anemic, while 47% of children under five in Bankura suffer anemia (5). Iron deficiency anemia is the most critical form affecting these populations, severely impacting women's health, and leading to complications such as severe anemia in pregnancy, which contributes to maternal mortality. The Maternal Mortality Ratio (MMR) for West Bengal is 104 deaths per 100,000 live births (2021–23) (7), higher than the national average of 88, indicating ongoing health system challenges. Key causes include severe anemia, postpartum hemorrhage, eclampsia, and pregnancy-related infections, often worsened by micronutrient deficiencies (iron, folic acid, vitamin B12) intertwined with economic hardships faced by women. These deficiencies increase maternal health risks and perpetuate cycles of poor health, limiting growth and economic productivity across generations, imposing costs on their households, communities, and the broader economies of which they are a part (6).

Although the unified guidelines of the Anemia Mukt Bharat program are expected to be followed universally, a significant gap exists in the implementation and research concerning remote and marginalized areas like those inhabited predominantly by Scheduled Castes, Scheduled Tribes, and Other Backward Classes (SC, ST, OBC) families in geographically isolated villages of India. Currently, no existing literature comprehensively addresses a holistic anemia control approach tailored to such vulnerable populations, considering cultural norms and family dynamics that influence health service adherence within these contexts. This observed gap highlights the need for localized, inclusive research and intervention strategies aligned with the national framework but sensitive to socio-cultural realities.

## 2 Materials and methods

### 2.1 Study setting

A qualitative study was conducted in Bankura District, West Bengal, across four Gram Panchayats, to analyze the behavioral and societal factors influencing adherence to health services and the adoption of healthy nutritional practices. Focus group discussions (FGDs) were organized to explore community perceptions of how gender roles and stereotypes shape health and well-being. These sessions were held during Village Health and Nutrition Days (VHNDs) in empty rooms of Anganwadi Centres or Health Sub-Centres. The study location, being a largely rural and agricultural region with rich cultural heritage and diverse social groups—particularly Scheduled Castes and Tribes dependent on farming, provided valuable context. An initial period of observation helped the researcher understand local behaviors, gender dynamics, and community interactions with health services, which validated and refined the FGD guide to suit the field realities. Conducting these discussions in such remote rural settings offered unique insights into the lived experiences of participants, allowing for an in-depth understanding of the underlying social dimensions of nutrition and health practices.

### 2.2 Description of participants

These focus group discussions were conducted in 4 Gram Panchayats (refer to Table 1). Three groups of participants were included in this study. Pregnant women, lactating mothers, and their respective mothers-in-law from each village. The study included three groups of participants: pregnant women, lactating mothers, and their respective mothers-in-law, from four Gram Panchayats. The pregnant women (*n* = 17), aged 20–25, with educational qualifications ranging from middle school to higher secondary school. The lactating mothers (*n* = 11) aged 21–26, with educational backgrounds including middle school, high school, and higher secondary school. The mothers-in-law (*n* = 13) aged 49–56, with educational qualifications ranging from illiterate to middle school. To enhance clarity, the names of the four Gram Panchayats will be referred to as Village A, Village B, Village C, and Village D.

**Table 1:** Participant demographics in FGDs (*N* = 41).

**Groups**	**Gender**	**Age**		**Gram panchayat**		**Educational qualification**	
Pregnant women (*n* = 17)	Female	20–22	9	Village A	2	High school	7
		23–25	8	Village B	3	Higher secondary school	8
				Village C	5	Middle school	2
				Village D	7		
Lactating mothers (*n* = 11)	Female	21–23	5	Village A	2	High school	7
		24–26	6	Village B	3	Higher secondary school	2
				Village C	4	Middle school	2
				Village D	2		
Mothers-in-law (*n* = 13)	Female	49–52	5	Village A	2	Illiterate	5
		53–56	8	Village B	2	Primary School	4
				Village C	4	Middle School	4
				Village D	5		

### 2.3 Sampling

A purposive sampling approach was employed to select participants for the Focus Group Discussions (FGDs). In total, six FGDs were conducted—two each in Villages C and D, and one each in Villages A and B of Bankura District, West Bengal. Villages C and D were chosen for additional FGDs as they had larger populations and a higher number of Anganwadi Centres or Health Sub-Centres compared to Villages A and B. Participants included pregnant women, lactating mothers, and their respective mothers-in-law who voluntarily consented to take part in the study. All participants were enrolled and registered at the designated Anganwadi Centres and Health Centres, and were initially approached through Accredited Social Health Activists (ASHAs) and Anganwadi Workers (AWWs). This purposive yet inclusive selection ensured representation from diverse household and generational perspectives within the study population, capturing variations in health-seeking behaviour and nutritional practices across the communities.

### 2.4 Data collection

Between November and December 2023, six focus group discussions were conducted among pregnant women, lactating mothers, and their respective mothers-in-law (*N* = 41). A thematic approach was used in analyzing the qualitative data. Initially, all FGD transcripts were in Bengali and were translated into English by bilingual researchers familiar with the local dialects and cultural context. Efforts were made to retain local phrases and cultural subtleties that were notable to theme development. The fidelity of the transcription and translation was ensured through repeated reading and validation of the transcripts before analysis.

**Figure 1:**
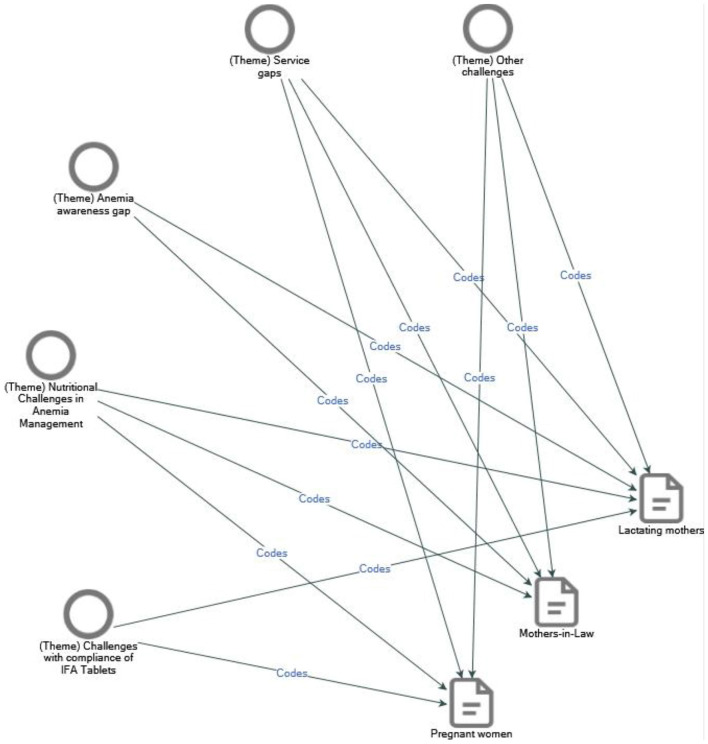
This diagram, prepared utilizing NVivo software, specifies that the codes assigned to each theme elicited responses of a multifaceted nature. For instance, the theme titled Nutritional Challenges in Anemia Management comprises three codes that align with the three distinct categories of participants involved in the Focus Group Discussions.

The transcripts were then collated and uploaded to NVivo 14.23.3. Relevant codes were developed inductively, and after several drafts, final coding was completed. These codes were subsequently categorized into themes aligned with the study objectives. Visualization of code and theme coverage was generated through the software (Figure 1). The assessment of the effectiveness of the anemia control program was demonstrated using verbatims mapped against each theme. All findings have been presented in a theme-driven format.

The researchers followed the pattern of the qualitative study conducted by Sedlander et al. (8) in Odisha, adapting the questions for the present context and validating them prior to data collection.

Multiple methods were used to ensure quality in this qualitative investigation. Methodological triangulation was employed across participant groups and existing literature to enhance credibility and validity. Data were compared between pregnant women, lactating mothers, and mothers-in-law across different villages to identify convergent and divergent patterns. Newly emerging themes were further triangulated with existing research, particularly the qualitative study by Sedlander et al. (8) conducted in Odisha. This comparative process enabled the researchers to assess whether the patterns observed in the field setting aligned with established determinants of IFA consumption and maternal nutrition practices. Several themes, including dislike of IFA taste, low compliance due to concerns about side effects and forgetfulness, increased household workload shaped by gender norms, and the influential role of mothers-in-law, were consistent with Sedlander et al.'s findings. These consistencies strengthen the reliability and transferability of the results by situating them within evidence from a similar socio-cultural context.

Peer review and validation were ensured through independent review of transcripts, codes, and theme summaries by multiple team members to establish equivalence and consistency. Member checking was conducted with a selection of participants who were contacted to provide feedback on summary interpretations, helping verify that the analysis accurately reflected their experiences. An audit trail was maintained through NVivo software, which provided a systematic and transparent record of coding decisions and theme development throughout the analytic process.

### 2.5 Ethical approval

This study has been approved by the Institutional Ethics Committee for Human Research (IEHCR), and the ethical approval number IEHCR/FCSc/M.Sc./10/2023/30 has been given. All participants provided written informed consent.

## 3 Results

The findings from the focus group discussions conducted in the Bankura District, focused on the fulfilment of the objectives of the study. It enabled us to understand the program effectiveness and identification of key barriers in the implementation of Anemia Mukt Bharat, including compliance with IFA supplementation, service delivery by field-level functionaries, and supply logistics management. All the findings have been demonstrated in a theme-driven format. Verbatims have been included, relevant to each theme.

Before beginning with the dissemination of the qualitative findings, it was crucial to know that any of the participants have ever heard about anemia or “*Roktalpota*” (in Bengali). The community related “*Roktalpota”*/anemia as a health condition where “*blood levels decreased*”. The health workers use the same terminology when speaking to them.

### 3.1 Nutritional challenges in anemia management

Lower maternal hemoglobin, aversion towards iron and folic acid-rich foods like green leafy vegetables, as well as throwing away the fortified rice kernels of the fortified rice, procured from the ration shop prove to be some of the important aspects or codes developed under this theme in understanding the nutritional behaviors and their impact within the community.

The results of nutritional gaps revealed, among all of the Gram Panchayats, a dislike in taste of locally grown iron and folic acid-rich foods like pumpkin leaves and “*Kulekhara”* saag (Hygrophila), among others, was found to be more prevalent among pregnant women than lactating mothers.

“*I have no difficulty preparing dishes from green leafy vegetables. I cook them every day, but my daughter-in-law doesn't have them at all,”* says a 45-year-old mother-in-law.

With the abundance of green leafy vegetables that are locally grown in the villages, the mothers-in-law complained of the aversion towards green leafy vegetables by their respective daughters-in-law.

Through the social safety net programs, fortified rice was being provided as a part of regular meals by ICDS centers to pregnant women and lactating mothers, as well as through the PDS, from the ration shop. Most of the mothers-in-law and lactating women, were throwing away the kernels. The communities identified the fortified rice as “*Protein Chaal/Vitamin Chaal.” (Protein rice/Vitamin rice)*.

**Figure 2:**
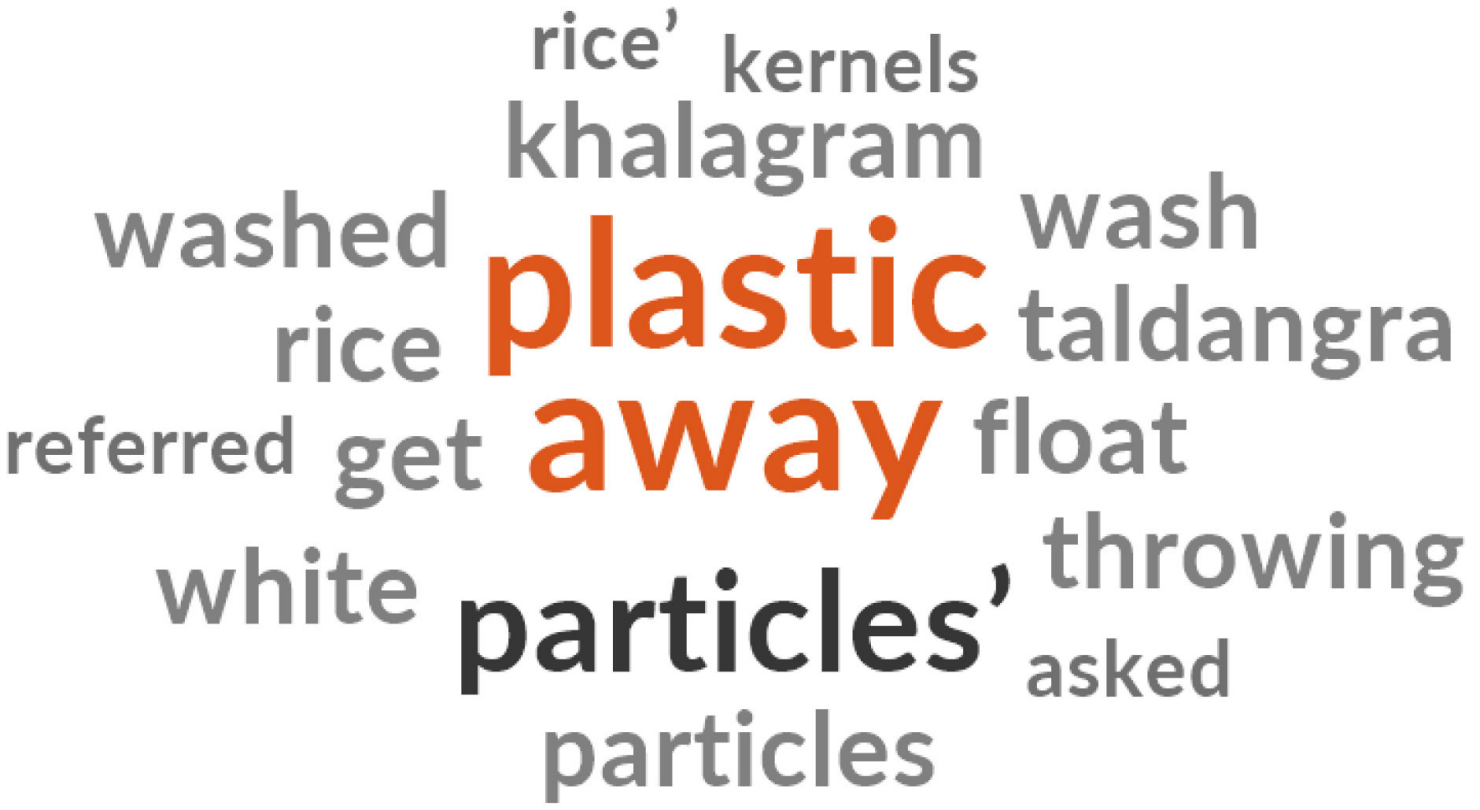
A word cloud, (generated from the NVivo Software) representing the most usage of the words when expressing their experience with fortified rice.

As it is observed from the word cloud, the participants referred to the word “plastic” (Figure 2) when referring to the appearance of the fortified rice kernels.

“*The white particles in the rice look like plastic. So, we throw them away.”* a 50-year-old mother-in-law. Most of the mothers-in-law and lactating women in all four Gram Panchayats referred to the kernels as plastic rice, which they threw away.

“*My daughter-in-law has a blood level of 7.2. She takes the tablets daily. She just got it checked today. But she doesn't have any green, leafy vegetables at all. She refuses to have green, leafy vegetables. Now that she's had it for one week, she was counselled by the ASHA/ANM didi, even today as well, as her blood levels were very low*,” says a 49-year-old mother-in-law.

All of the pregnant women from the four Gram Panchayats reporting low hemoglobin levels ranged from 7.2 being the lowest to 11 being the highest. The ANMs (Auxillary Nurse Midwives) check their hemoglobin levels, during their ANC visit.

### 3.2 Challenges with compliance with IFA tablets

Understanding the behaviors or factors that contribute to non-compliance is crucial in tackling programmatic challenges related to IFA tablet/*Boris* (in Bengali) compliance among the women in the respective communities. Based on the results output, disliking the taste of the tablets, forgetting to consume the tablets or *Boris*, and other challenges/issues faced in taking the IFA tablets are some of the components that have been used to understand this theme.

“*I force myself to take the boris”. “I don't like taking the boris at all. It doesn't have good taste. Having the boris will increase the blood level, but the aftertaste of the boris is very bad,”* says a 22-year-old pregnant woman.

Despite knowing the benefits of taking the tablet, many pregnant women and lactating mothers in all the Gram Panchayats have opined against the taste of the IFA tablets. Thus, it is understood that the taste is a hindrance for compliance.

“*Due to the bad taste of the tablets, I don't like taking them at all. I force myself to take them. Sometimes I miss taking them because of the taste,”* says a 23-year-old pregnant woman from Village B. From this statement, we could understand the taste was a major factor in the compliance of IFA.

“*I feel nauseous after taking the tablet. There's a weird smell I get when I have the boris,”* says a 21-year-old pregnant woman.

Due to the tablet, a few pregnant women from this panchayat have complained of feeling nauseous, which has stopped them from taking the tablet.

**Figure 3:**
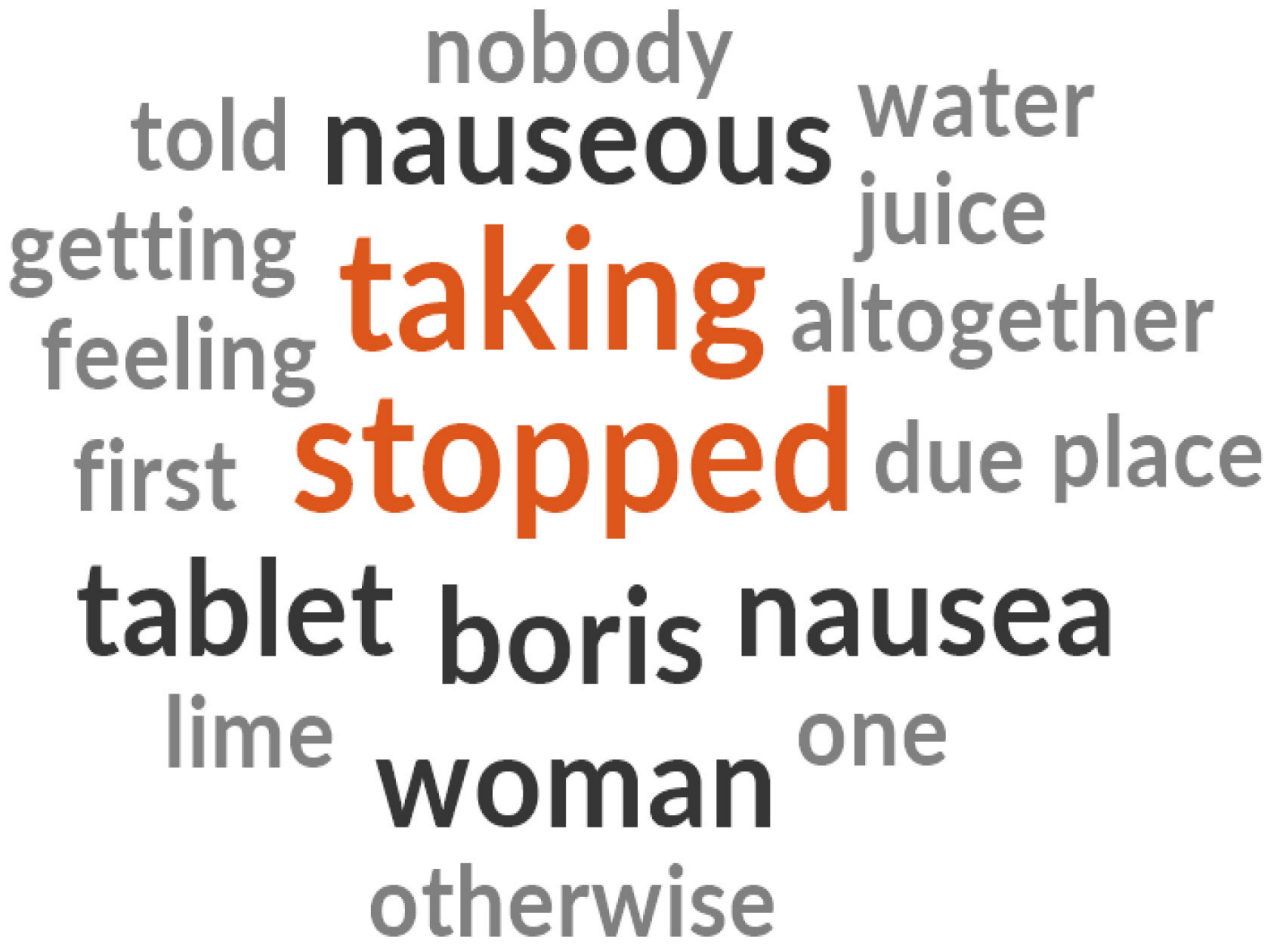
A word cloud, (generated from the NVivo Software) representing the most usage of the words when expressing their other challenges with IFA compliance.

As observed, the participants referred to the word “taking, stopped, nausea” (Figure 3) when referring to the nauseated experience with intake of IFA tablets.

Another aspect of the theme was forgetfulness—“*Most of the time, we forget to take these tablets on time as we have a lot of work at home,”* says a 24-year-old lactating mother. While discussions happened, most of these pregnant and lactating women of all gram panchayats opined of forgetting to take the tablets on time, reasoning attributed to lot of household work.

“*I forget so many things because of old age. I can't remind my daughter-in-law to take her IFA tablets or Boris. I sometimes forget to take my medicines. My daughter-in-law reminds me to take my medicines on time*,” says a 56-year-old mother-in-law.

The mothers-in-laws maintain that they are unable to remind their daughters-in-law to take their medicines on time due to their old age. Majority of their husbands either reside elsewhere in search of work or are too preoccupied with work to assist their wives in reminding their daughters-in-law to take their IFA tablets. So, pregnant and lactating women remind themselves to take the tablets, and opined as forgetfulness as a major reason.

### 3.3 Anemia awareness gap

To comprehend the behaviors and practices related to the use of IFA tablets, it is crucial to understand the community's awareness on anemia, IFA consumption. The theme also considers the forgetfulness of lactating mothers regarding IFA intake.

“*I am not aware of what happens after the tablet is taken,”* says a 55-year-old mother-in-law.

The mothers-in-law across all the Gram Panchayats except for Village B were not aware of the benefit of taking the tablets and the symptoms when the “blood levels” decreased in one's body.

“*No. I am not aware of these issues faced by the women consuming the bori,”* says a 52-year-old mother-in-law.

The mothers-in-law were unaware of the challenges that their pregnant and lactating daughters-in-law faced while taking their IFA tablets. Neither of these women reminded their daughters-in-law to take their tablets on time, and they also assigned most of the household chores to them.

It was observed that the lactating mothers had no recollection of information related to anemia. When asked about the symptoms of anemia or when the “blood levels decrease”, they responded that they didn't know.

“*I have forgotten most of the things. When I was pregnant, the ASHA didis told us to have the IFA Boris as it would increase our blood levels,”* says 24-year-old lactating mother.

The participants were not aware of who the beneficiaries of the IFA tablets were. Most of them thought that only pregnant women and lactating mothers received the tablets from the sub-centre, as well as the ASHA (Accredited Social Health Activists) *didis*. A few of the pregnant women and lactating mothers knew that school-going adolescents also received the tablets from their respective schools. However, they were not aware that non-pregnant and non-lactating women also received the tablets. They claimed that women who are non-pregnant and non-lactating do not receive the tablets from the sub-center.

One of the husbands of the pregnant women who participated in the discussion was neither aware of the symptoms of anemia nor the issues faced by women when taking the tablets.

### 3.4 Service gaps

The front-line workers, mainly the ASHA *didis* in the respective villages, are responsible for the timely provision of IFA tablets to all the beneficiaries, along with appropriate counselling on IFA tablet consumption and intensive awareness generation on anemia for not only pregnant mothers but all the beneficiaries of the program.

“*I have forgotten most of the things. When I was pregnant, the ASHA didis told us to have the IFA Boris as it would increase our blood levels,”* says 22-year-old lactating mother.

Across all four Panchayats, it was found that most lactating mothers had limited knowledge about anemia, including its risk factors and consequences.

Through the discussions, it was observed that limited anemia counselling on the dosage of the tablet and the reasons for taking the tablets, especially during the critical periods of pregnancy and lactation, are not explained effectively to these mothers by the front-line workers.

“*I get information on the timing of consuming the IFA tablet/boris. I don't know about the dosage or the reasons for taking the tablet. I know that it will help me raise my blood levels. But I don't know why I am taking the tablets in the first place,”* says a 22-year-old pregnant woman. This revealed a significant service gap in IFA tablet counseling, by the health functionaries, across all Gram Panchayats.

“*I have stopped taking them within a month after having the baby. I have not been given any boris thereafter. I still have a few of them left, so I take them once a day,”* says a 25-year-old lactating mother.

This mother, along with other lactating mothers in the discussion at Village B, did not receive tablets after their pregnancy, despite being beneficiaries of the anemia control program. They had completely stopped taking the tablets and were not even aware that they should be receiving them in the first place.

“*I had to procure the boris from outside, as the ASHA didi told me that there was less stock of boris at the sub-center,”* says a 22-year-old pregnant woman.

Through the discussions, it was observed that the health sub-center in this panchayat had issues with the supply of IFA tablets and was asked to purchase the tablets from a pharmacy instead of receiving them for free, from the respective primary health centers, and sub-centers as per the program guidelines.

“*I once had an issue regarding my periods before my pregnancy. I was having irregular periods. Women who are not pregnant and not lactating don't get any tablets from the sub-center,”* says a 22-year-old lactating mother.

According to participants in discussions across all Gram Panchayats, women of reproductive age are not receiving their entitled IFA tablets from health centers. These women are also not aware that non-pregnant and non-lactating women should receive tablets from the sub-centers once a week.

### 3.5 Other challenges

This theme has been incorporated in this study to analyze the societal components of gender discrimination and the increased burden on women for household work in these communities, which cannot be ignored. These components aspects are integral to understanding the deep-rooted gender stereotypes, which should be studied further to understand the community and address the “nutrition-sensitive” challenges of anemia.

“*I do most of the household chores. My mother-in-law is old, so she won't be able to help me out. My husbands also don't live here; therefore, I must do all the household work,”* says a 20-year-old pregnant woman.

All the participants in the discussions across all the panchayats claim that they do all the household work and don't ask men to help them out. Since most of their husbands live outside, and their mothers-in-law are old, the pregnant and lactating women of Villages C and D do all the household chores. In contrast, the pregnant and lactating women of Villages A and B receive help from their mothers-in-law to do the household chores.

“*Most of the time, the women forget to take these tablets on time as we have a lot of work at home,”* says a 24-year-old lactating mother.

Most pregnant women and lactating mothers have claimed, from all the Gram Panchayats, that they have a lot of household work, which makes them forget to comply with the intake of tablets regularly.

“*I forget so many things because of old age. I can't remind my daughter-in-law to take her IFA. I sometimes forget to take my medicines. My daughter-in-law reminds me to take my medicines on time,”* says a 56-year-old mother-in-law.

The mothers-in-law residing in the Villages C and D Panchayats maintain that they are unable to remind their daughters-in-law to take their medicines on time due to their old age. The majority of their husbands either reside elsewhere or are too preoccupied with work to assist their wives in reminding their daughters-in-law to take their IFA tablets.

So, pregnant, and lactating women remind themselves to take the tablets, complaining of forgetting to take them at the same time.

“*I think that women are treated differently. Men go and work outside. The women in our community stay at home and do work”*, says a 21-year-old lactating mother.

As observed, the participants claimed that gender differences persist, where men work in cities while women work in households. They have not witnessed women working outside the city as earning members of the household. During discussions, mothers-in-law assert that women work at home while men earn. This appears paradoxical, as they claim to see no differences between men and women despite the statements.

“*If families have the money to help the girls pursue their higher education, only then is it possible. Having money to pursue higher education is crucial. Most of the women here have completed their 10th and 12th grades in school,”* says a 22-year-old pregnant woman.

“*Few of them drop out of school and get married. Many girls try to run away and get married. There's pressure on the family's side for the girl to get married early as well,”* says a 23-year-old pregnant woman.

## 4 Key findings

- The community does not recognize “anemia,” instead referring to it as “blood levels decreasing.”
- Pregnant women often avoid iron- and folic acid-rich foods like green leafy vegetables, sometimes due to nausea, while many discard fortified rice kernels, mistaking them for plastic.
- Compliance with iron-folic acid (IFA) tablets is affected by their taste, forgetfulness amid heavy workloads, and a lack of reminders from family.
- There is a significant awareness gap regarding anemia symptoms and the benefits of IFA tablets. Many women and their mothers-in-law lack proper knowledge and often forget counseling sessions.
- Service delivery is challenged by insufficient IFA tablet supplies, especially postnatally, and inadequate dosage counseling, leading to poor adherence.
- Postnatal care tends to focus on newborns, neglecting mothers' health.
- Socially, traditional gender roles place heavy burdens on women, limiting their compliance with health recommendations.
- Influences from mothers-in-law can hinder their daughters-in-law's nutrition, and the limited involvement of male family members weakens support systems for anemia prevention.

## 5 Discussion

In our country, the community's perceptions and expectations are that women should work outside as well as take care of household chores and manage their health issues, which are concerned with women's contribution to the community's development (9). Study participants expressed similar views, stating that daily household chores often led them to forget to take IFA tablets. The unequal distribution of household labor contributed to these women neglecting their own health, prioritizing the needs of other family members, and ultimately compromising their overall well-being. When women lack essential vitamins and minerals, the consequences are severe. The men of the communities were working outside the city or were also in faraway places.

The observation that women in the community avoid consuming green leafy vegetables (GLVs) due to nausea, coupled with a shallow perception of anemia risk, is consistent with findings in other rural and low-resource settings in India. Studies have shown that poor dietary diversity and low intake of iron-rich foods often stem from both physiological factors like nausea during pregnancy and deeply ingrained food preferences or taboos. Moreover, limited community education and inadequate counseling on anemia contribute to low awareness and underestimation of its health risks, which has been reported widely in similar socio-cultural contexts (10). These parallels highlight the critical need for context-specific, culturally sensitive nutrition education and behavior change communication to effectively address misconceptions and promote anemia prevention through better dietary practices. This was also because there was no appropriate community education rendered to them. None of the participants, be they from health or ICDS as functionaries or community health workers, saw any social consequences of the consumption of IFA. The health workers in the community have not given adequate attention or counselling regarding anemia. To address this problem, health workers must provide comprehensive counselling on anemia and implement the existing anemia control programs effectively, providing timely interventions as per the program. There were multiple issues about the supply chain and logistics. Upon distribution, the tablets were prioritized for pregnant women, followed by women of reproductive age. Therefore, these women/Women of Reproductive Age (15–49 years) had to get back to the dispensary for more stocks. This was a major constraint due to the inbuilt roles and communicative distance. Mostly, it was opined by the participants that no specific instructions were given for the reason to consume IFA, nor was any specific information ever shared. Women's higher education is not given any importance in these communities. This becomes crucial for young women to be empowered and have the reproductive choice of having their children whenever they want.

Empowered women will exert better control over their healthy lifestyle and emotional well-being. According to the World Bank, “better-educated women tend to be more informed about nutrition and healthcare, have fewer children, [and] marry at a later age, and their children are usually healthier, should they choose to become mothers. They are more likely to participate in the formal labor market and earn higher incomes.” Empowering women while investing in their improved nutrition will have a multiplying effect on the resilience of women, their families, and their communities (9).

Global disparities in anemia reflect the significant differences that exist between developing and developed nations, as well as the variations in exposure to the various determinants of anemia. Developing countries are at a disadvantage when it comes to sufficient health care, nutrition, and amenities because of several societal factors that impact the most vulnerable in resource-limited settings. Along with environmental factors and other health determinants, public health plays a major role in understanding and resolving disparities in health outcomes, including disparities in health care access and quality (5). Effectively addressing disparities in healthcare and health requires a collective effort that involves a wide array of stakeholders within the public health and healthcare systems. Public health agencies can play a crucial convening role, collaborating with organizations and sectors such as community groups, healthcare delivery systems, academia, businesses, and the media.

## 6 Conclusion

This study provides unique insights into the complex interplay between health access and the socio-cultural realities shaping women's nutritional status in rural West Bengal. Unlike conventional assessments that view anaemia as a purely biomedical or programmatic concern, this research uniquely merges the understanding of health-associated problems with the underlying societal and gender barriers that impede service utilization and dietary compliance. Conducted within a limited time frame and across four Gram Panchayats in the Bankura District, the study's small sample size and reliance on secondary haemoglobin data collected by frontline health workers may limit the generalizability of its findings. By focusing on pregnant and lactating women—a group with emerging and evolving nutritional needs, the study reveals how social hierarchies, household roles, and limited agency significantly influence health-seeking behaviour and adherence to anemia control interventions.

The findings demonstrate that poor compliance with iron and folic acid (IFA) tablets, inadequate intake of nutrient-rich foods, insufficient counselling, and limited supply of supplements are compounded by gender inequities and social norms. These persistent challenges highlight the need for community-centered and gender-sensitive strategies within ongoing programs such as *Anemia Mukt Bharat*.

The study advocates for a life-cycle approach to anemia prevention—integrating nutrition-sensitive and nutrition-specific interventions while strengthening the capacity of frontline workers and involving key household members, particularly mothers-in-law and husbands, in awareness and counselling. At the policy level, enhancing multi-sectoral convergence between health, nutrition, and social protection sectors is essential to address these interlinked barriers. By intertwining social context with health system realities, this study contributes a nuanced understanding of how societal structures shape women's health access and outcomes. Such evidence can guide policymakers and practitioners toward more equitable, participatory, and gender-transformative approaches in maternal and child health programming across rural India (8, 9, 11).

## Data Availability

The original contributions presented in the study are included in the article/supplementary material, further inquiries can be directed to the corresponding authors.
